# A Randomised, Placebo-Controlled, Crossover Study Investigating the Effects of Nicotine Gum on Strength, Power and Anaerobic Performance in Nicotine-Naïve, Active Males

**DOI:** 10.1186/s40798-016-0074-8

**Published:** 2017-01-13

**Authors:** Toby Mündel, Marine Machal, Darryl J. Cochrane, Matthew J. Barnes

**Affiliations:** School of Sport and Exercise, Massey University, Private bag 11 222, Palmerston North, 4442 New Zealand

**Keywords:** Chewing gum, Doping, Stimulant, Performance, WADA

## Abstract

**Background:**

Nicotine use amongst athletes is high and increasing, especially team sports, yet the limited previous studies investigating the performance consequences of this behaviour have not examined the effects of the principal active ingredient, nicotine, per se. Therefore, we determined whether nicotine gum affected muscular and anaerobic performance.

**Methods:**

Nine active males (24 ± 3 years) completed three trials in a random order in which 20 min prior to testing they chewed 2 mg (NIC-2), 4 mg (NIC-4) nicotine or flavour-matched placebo (PLA) gum. Peak and average peak isometric, concentric and eccentric leg extensor torque was measured followed by vertical counter-movement jump height and a 30-s Wingate test. Heart rate was measured whilst capillary blood samples determined pH, HCO_3_^−^ and venous blood confirmed the presence of nicotine.

**Results:**

Nicotine was confirmed by the presence of its major metabolite, cotinine and participants reported no side effects with nicotine. Peak and average peak isometric and eccentric torque was significantly affected (NIC-2 > PLA; *p* < 0.05) whilst peak (NIC-2 > PLA; *p* < 0.05) but not average peak (*p* > 0.05) concentric torque was different between trials. Counter-movement jump height was similar across trials (*p* > 0.05). Anaerobic capacity during the Wingate remained similar across trials (*p* > 0.05); however, pacing strategy (peak power and rate of fatigue) was different during NIC-2 than PLA. pH was affected by nicotine (NIC-2 > PLA; *p* < 0.05) and was reduced following the Wingate in all trials. HCO_3_^−^ showed similar responses across trials (*p* > 0.05) although it was also reduced following the Wingate (*p* < 0.05), whilst heart rate was significantly affected (NIC-2/NIC-4 > PLA; *p* < 0.05).

**Conclusions:**

Chewing low-dose (2 mg) nicotine gum 20 min prior to exercise significantly improved leg extensor torque but did not affect counter-movement jump height or Wingate performance compared to a placebo, whilst there were minimal effects of the 4 mg nicotine gum on the performance parameters measured.

## Key Points

- Recent monitoring (urine screening) has determined that active consumption of nicotine and nicotine-containing substances in-competition occurs in approximately 25–50% of athletes in sports that are characterised by strength, power and anaerobic capacity (cf. endurance), e.g. American football, ice hockey, wrestling, bobsleigh, gymnastics, rugby and skiing.
- Whilst the World Anti-Doping Agency placed nicotine onto its monitoring program in 2012, few studies have determined the performance effects of nicotine administration, and none without the confounders of tobacco, withdrawal and tolerance.
- The current study has demonstrated that a low-dose (2 mg) nicotine gum increases leg extensor torque but counter-movement jump height and anaerobic capacity remained unchanged when compared to a placebo gum.
- These and our previous results indicate that nicotine per se can improve exercise endurance and muscular strength, and alongside patterns of (mis)use future studies should continue to practically and mechanistically determine the full extent of this under-researched stimulant in terms of performance enhancement and athlete health.

## Background

The (ab)use of nicotine and nicotine-containing substances by athletes, especially by professional and elite status, is prevalent. For example, Marclay et al. [20] reported on a one-year monitoring study of 2185 urine samples from professional athletes in 43 different sport disciplines. Traces of nicotine and/or tobacco-related alkaloids were detected in 23% of the samples, with prevalence of “active” nicotine consumption (cf. passive environmental exposure) immediately prior to and/or during sport practice determined at 15% [20]. Of note, cumulative exposure of >25% (greater than the worldwide prevalence in the general population, as reported by the World Health Organization) was reported in American football (56%), ice hockey (32%), wrestling (32%), bobsleigh (31%), gymnastics (29%), rugby (28%) and skiing (26%). These findings were preceded by the alarming observation that approximately half of the athletes at the 2009 Ice Hockey World Championships were active nicotine consumers before and/or during games [21]. Therefore, the use of nicotine by athletes, regardless of its form (e.g. snuff, snus), is frequent across many sports and countries (e.g. [8, 22]).

Athletes’ beliefs are that consumption of nicotine/smokeless tobacco proves ergogenic by preventing xerostomia [6], weight control [2], improving reaction time and concentration [13], helping relaxation and desirable arousal-attention [8]. These reports are supported by a meta-analysis determining that the stimulant effects of nicotine enhance aspects of cognition and attention, namely motor abilities, attention and memory [15]. The results are noteworthy as included studies were not confounded by nicotine’s withdrawal effects, lack of a placebo control, patient group deficit or elderly decline in cognition and therefore likely represent true performance enhancement. However, there are reports that a dose–response relationship exists whereby lower doses prove nootropic whilst higher doses do not [25, 26], this is perhaps due to the known pharmacological and physiological action of low-dose nicotine as a central nervous system (CNS) stimulant whilst at high doses, a depressant or relaxant effect occurs [18, 28].

As the sports and athletes with the most use of nicotine concern strength, power and anaerobic events (see above), it follows that performance and research protocols reflect this. Previous research has shown no performance enhancement with smokeless tobacco use for leg extensor force and power [12], anaerobic performance [2, 27] or handgrip strength, counter-movement jump and agility [23]. However, smokeless tobacco includes many other ingredients that might confound results and the use of participants that are habitual users is confounded by withdrawal or tolerance effects, whether acute or chronic. In an effort to determine the actual performance effects of nicotine in an acute experimental setting, in the present study, nicotine-naïve participants received acute nicotine supplementation, as using this rationale, we and others have previously demonstrated physical and cognitive performance enhancement [15, 24] *despite* the oft-reported side effects [23, 24].

Nicotine was added to the World Anti-Doping Agency (WADA) Monitoring Program in 2012, indicating that WADA wished to detect patterns of misuse [29]. Concurrently, there should be an increased investigation focusing on whether nicotine can enhance performance and/or represents a health threat to athletes in order to inform WADA on whether nicotine should remain a monitored substance or even be upgraded to the List of Prohibited Substances. Therefore, the experimental hypothesis of the present study was that through its stimulatory effect on the CNS, nicotine would prove ergogenic at the lower but not at the higher dose administered compared to a placebo for measures of leg extensor torque, muscular power and anaerobic performance.

## Methods

### Participants

Nine healthy, never-smoker males were recruited for the study (mean ± SD: age, 24 ± 3 years; body mass, 78 ± 15 kg; height, 179 ± 13 cm). All participants had been competitive in team sports and trained (including gym sessions) or competed regularly ≥3 times per week for at least 2 years. Each participant was fully informed of all potential risks and experimental procedures, after which informed written consent was obtained. All experimental procedures and protocols were approved by the Institutional Human Ethics Committee and performed in accordance with the latest revision of the Declaration of Helsinki.

### Study Design

**Fig. 1:**
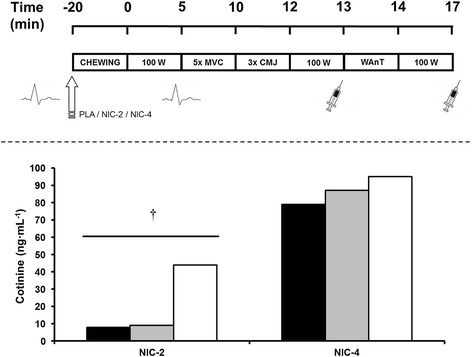
*Upper panel*: Schematic of experimental protocol. Seated rest for 5 min and HR measurement followed by chewing gum for 20 min, a cycle ergometer warm-up at 100 W and another HR measurement. A set of five MVC’s each for ISO, CON and ECC leg extensor force separated by a 2-min rest. Three maximal CMJ, after a further 1-min cycling at 100 W a capillary blood sample followed by the WAnT and 3-min cool-down at 100 W ending with a capillary blood sample. *Lower panel*: Individual plasma cotinine concentration in 2 mg (NIC-2) and 4 mg (NIC-4) gum trials for the third, sixth and ninth participants to start trials; none detected for PLA. ^†^Significant difference to corresponding NIC-4 value

All testing was conducted in the same laboratory environment (~20 °C, 600 lx). Participants visited the laboratory on four occasions, one anthropometric and familiarisation session and three experimental trials separated by > 2 < 7 days. All participants had previously completed the measures of performance required for this study; however, a full familiarisation was still performed before commencing the experimental trials to minimise learning. Experimental trials were conducted at the same time of day (±1 h), and the day of and prior to any experimental trial was marked by abstinence from alcohol, any exercise and only habitual caffeine use (as abstinence would in itself confound from withdrawal effects). Additionally, participants were asked to replicate their diet during the first experimental visit for subsequent trials to ensure a similar metabolic state. For experimental trials, 30 min prior to performance testing, participants chewed 2 mg (NIC-2) or 4 mg (NIC-4) nicotine or a flavour-matched placebo (PLA) gum, the order of which was randomised using a Latin square design. Immediately following this, participants completed five voluntary maximal contractions (MVC) each of isometric (ISO), concentric (CON) and eccentric (ECC) contractions of the *quadriceps femoris* muscle of the dominant leg followed by three maximal vertical counter-movement jumps (CMJ) and a 30-s Wingate test (WAnT). A 5-min ergometer warm-up at 100 W preceded the measurement of leg extensor force and a further 1-min warm-up prior to the WAnT. This order of tests was kept constant and was <20 min in duration (Fig. 1).

### Experimental Protocol and Measures

On arrival to the laboratory, participants fastened a heart rate (HR) strap and monitor (Polar, Kempele, Finland) and were seated for 5 min, after which a baseline HR measurement was taken. Participants then chewed gum for 20 min whilst seated before completing a 5-min warm-up on a cycle ergometer (Monark, Varberg, Sweden) at 100 W, during which HR measurement was taken in the final minute before removing the gum. The participant was then seated on the isokinetic dynamometer (Biodex Medical Systems, New York, USA) at the previously recorded seat adjustments so that the femoral epicondyle was aligned with the dynamometer’s axis of rotation and the ankle strap positioned 5 cm proximal to the medial malleolus. Along with the ankle, straps were placed around the chest, the hips and the leg to be tested in order to isolate the *quadriceps femoris* muscle. The lower limb was weighed, with the knee fully extended, to account for the action of gravity. CON and ECC contractions were performed over a 60° range of motion (from ~110° knee flexion) and ISO tension measured at 75°. The participant then performed five MVC of each type with each set separated by 2 min of passive recovery. CON and ECC torque was measured at an angular velocity of 30° s^−1^. Peak and average peak torque for CON and ECC, and peak and average peak ISO tension (average calculated from the five contractions) were recorded.

Participants then performed three CMJ, each separated by 30 s of passive rest. Participants descended to a self-selected depth and immediately jumped upward as high as possible. To exclude the influence of arm swing, participants were instructed to keep their hands placed on their hips. Jump height was measured with an electronic jump mat (Swift Performance, NSW, Australia), and the peak and average (of three) values were recorded. Following this, participants completed a further 1-min cycling at 100 W during which a 50-μL fingertip blood sample was collected into a heparinised glass capillary tube, before completing their WAnT. The WAnT was completed on a friction-loaded ergometer (Monark, Varberg, Sweden) using a resistive load of 7.5% of body mass, where cadence was recorded every 5 s and power output calculated from friction load and flywheel velocity. The highest and lowest 5-s values were used to determine peak power (W) and rate of fatigue (%), whilst the sum of all 5-s values determined the anaerobic capacity (kJ). Finally, participants cooled down for 3 min at 100 W at which time collection of a final 50-μL fingertip blood sample occurred. The capillary blood samples were analysed immediately for determination of pH and bicarbonate (HCO_3_^−^) via an automated analyzer (Radiometer, Brønshøj, Denmark).

### Nicotine Intervention and Verification

Participants were instructed according to the manufacturer’s recommendations: one piece of gum (2 mg, 4 mg and placebo; Nicorette Icy Mint, Johnson & Johnson Pacific, Auckland, New Zealand) was introduced into the mouth followed by the instructions “chew until there is a strong taste, then place between your cheek and gums, and chew again when the taste has faded”, with this encouraged for 20 min. Participants were not aware of the research hypotheses, only that on two of the three occasions they would be receiving nicotine. To verify the presence of systemic nicotine a resting (following 20-min chewing but prior to warm-up) venous blood sample was obtained from every third participant beginning the study (i.e. *n* = 3); this reduced sample was due to resource limitation. Venous blood samples were obtained from an antecubital vein into a 4-ml lithium heparin vacutainer tube (Becton–Dickinson, Plymouth, UK) then placed on ice for 10 min before being centrifuged (Eppendorf, Hamburg, Germany) at 4 °C for 10 min at 805*g*. Plasma was removed, aspirated into 500-μl aliquots and frozen at −80 °C for later analyses using high-performance liquid chromatography (HPLC). Due to nicotine’s tendency to fluctuate and relatively short half-life (~2 h), cotinine, its major (~70%) metabolite with a longer retention time (~18–20 h) is preferred [11]. Sample preparation, solid phase extraction and analysis by HPLC were based on previous methodology [9] and performed in duplicate.

### Statistical Analysis

All statistical analyses were performed with SPSS software for windows (IBM SPSS Statistics 20, NY, USA). Descriptive values were obtained and reported as means and standard deviation (SD) unless stated otherwise. Levene’s test was used to ensure that data did not differ substantially from a normal distribution. Baseline/resting data (for physiological variables) were first analysed using one-way ANOVA. Data repeated over time (pH, HCO_3_^−^, HR) were analysed by two-way (trial × time) ANOVA, whilst all other (performance) data were analysed by one-way ANOVA. Sphericity was assessed and where the assumption of sphericity could not be assumed, adjustments to the degrees of freedom were made (ε > 0.75 = Huynh-Feldt; ε < 0.75 = Greenhouse-Geisser). Following a significant *F* test post-hoc pairwise analyses were performed using a paired samples *t* test (Bonferroni correction where relevant), with statistical significance set at *p* ≤ 0.05. Partial eta-squared (*η*_p_^2^) is reported as a measure of effect size, with demarcations of small (<0.09), medium (>0.09 < 0.25) and large (>0.25) effects.

## Results

No order effects were observed (all *p* > 0.11, *η*_p_^2^ < 0.16) and all participants completed the study without reporting negative side effects; the only comment made was that most experienced a scratchy/tickly throat during some (nicotine) trials.

### Physiological Variables

Of the samples screened for blood cotinine concentration (*n* = 3; Fig. 1), none was detected during PLA whereas cotinine concentration increased as a function of dose (NIC-2: 20 ± 21 ng mL^−1^, NIC-4: 87 ± 8 ng mL^−1^; trial: *p* = 0.01, *η*_p_^2^ = 0.97). Resting HR was similar prior to treatment (trial: *p* = 0.49, *η*_p_^2^ = 0.08) but following treatment differed between trials as a function of time (trial × time: *p* = 0.03, *η*_p_^2^ = 0.34; Table 1) such that the increase in HR was more pronounced during NIC-2 (6 ± 7 beats · min^−1^) and NIC-4 (7 ± 9 beats · min^−1^) compared to PLA. HCO_3_^−^ before the WAnT was similar between trials (trial: *p* = 0.40, *η*_p_^2^ = 0.11) but was decreased following the WAnT (time: *p* < 0.01, *η*_p_^2^ = 0.97), whilst pH was different between trials (trial: *p* = 0.03, *η*_p_^2^ = 0.37) and across time (time: *p* < 0.01, *η*_p_^2^ = 0.90).

**Table 1:** Heart rate, venous bicarbonate and pH for placebo, 2 and 4 mg nicotine gum. Values are mean ± SD, *n* = 9

	Pre			Post		
	PLA	NIC-2	NIC-4	PLA	NIC-2	NIC-4
HR (beats min^−1^)	65 ± 11	67 ± 9	64 ± 9	117 ± 15^a^	125 ± 19^a,b^	123 ± 15^a,b^
HCO_3_^−^ (mmol L^−1^)	25 ± 5	27 ± 2	25 ± 5	14 ± 4^a^	15 ± 3^a^	16 ± 4^a^
pH	7.46 ± 0.08	7.52 ± 0.06^b,c^	7.47 ± 0.06	7.29 ± 0.10^a^	7.35 ± 0.08^a^	7.30 ± 0.10^a^

Heart rate (HR) at rest pre-treatment (Pre) and during cycling at 100 W post-treatment (Post); bicarbonate (HCO_3_^−^) and pH before (Pre) and after (Post) WAnT; placebo (PLA), 2 mg (NIC-2) and 4 mg (NIC-4) trials

^a^Significant difference to corresponding Pre value

^b^Significant difference to corresponding PLA value

^c^Significant difference to corresponding NIC-4 value

### Performance Variables

**Fig. 2:**
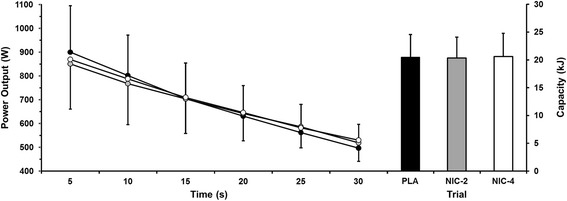
Power output and anaerobic capacity during the WAnT for placebo (*filled circle*, PLA), 2 mg (*grey circle*, NIC-2) and 4 mg (*open circle*, NIC-4) nicotine gum. Values are mean ± SD, *n* = 9

Peak and average ISO tension differed between trials (trial: *p* = 0.02, *η*_p_^2^ > 0.37; Table 2) such that NIC-2 > PLA. Peak (trial: *p* = 0.05, *η*_p_^2^ = 0.30) but not average (trial: *p* = 0.28, *η*_p_^2^ = 0.15) CON torque differed between trials such that NIC-2 > PLA. Peak (trial: *p* < 0.01, *η*_p_^2^ = 0.48) and average (trial: *p* = 0.03, *η*_p_^2^ = 0.37) ECC torque differed between trials such that NIC-2 > PLA. Peak and average CMJ height was similar between trials (trial: *p* > 0.65, *η*_p_^2^ < 0.05). During the WAnT (Fig. 2), anaerobic capacity was similar between trials (trial: *p* = 0.73, *η*_p_^2^ = 0.04) although pacing strategy seemed to differ; peak power differed between trials (trial: *p* < 0.01, *η*_p_^2^ = 0.57) such that NIC-2 < PLA by 6 ± 3% or 49 ± 24 W whilst the rate of fatigue differed between trials (trial: *p* < 0.01, *η*_p_^2^ = 0.59) such that NIC-2 (38 ± 11%) and NIC-4 (37 ± 12%) < PLA (44 ± 10%).

**Table 2:** Peak and average leg extensor torque and counter-movement jump height for placebo, 2 and 4 mg nicotine gum. Values are mean ± SD, *n* = 9

	Peak			Average		
	PLA	NIC-2	NIC-4	PLA	NIC-2	NIC-4
Leg extensor torque (N m)						
ISO	309 ± 79	330 ± 82^a^	314 ± 79	290 ± 73	309 ± 77^a^	293 ± 71
CON	240 ± 59	258 ± 67^a^	251 ± 53	208 ± 61	218 ± 66	220 ± 59
ECC	332 ± 77	356 ± 73^a^	350 ± 75	316 ± 81	334 ± 73^a^	332 ± 76
CMJ (cm)	38 ± 8	38 ± 9	37 ± 9	37 ± 8	37 ± 9	36 ± 9

*ISO* isometric, *CON* concentric and *ECC* eccentric torque; *CMJ* counter-movement jump; *PLA* placebo, 2 mg (NIC-2) and 4 mg (NIC-4) trials

^a^Significant difference to corresponding PLA value

## Discussion

This is the first study to investigate whether nicotine improves measures of leg extensor torque, muscular power and anaerobic performance. The main findings from this study were that (1) low-dose nicotine (2 mg) increased leg extensor force by ~6% compared to the placebo, whereas neither low- nor high-dose nicotine affected counter-movement jump height; (2) during the WAnT, anaerobic capacity was unchanged although low-dose nicotine altered the pacing strategy adopted; (3) physiologically, low-dose nicotine caused a relative alkalosis and both nicotine doses caused heart rate to be higher than placebo.

### Low-Dose Nicotine Increases Peak and Average Leg Extensor Torque But Not Muscular Power

Overall, five out of six measures of torque were significantly improved with NIC-2 (6 ± 5%) with only the average concentric torque unaffected (4 ± 4%). To our knowledge, only one previously published study has measured leg extensor performance. Escher et al. [12] tested a greater range of flexion (90°) and angular velocity (250° s^−1^) than in the current study using college athletes who regularly used smokeless tobacco; their participants were tested following ~12-h abstention from tobacco or having consumed smokeless tobacco 2 h and immediately prior to testing. They reported that MVC was lower when using compared to abstaining from tobacco but commented that they do not know whether it was the nicotine or any other substance within tobacco proving ergolytic. However, cotinine concentration during the tobacco trials in that study were considerably higher than in our study when using NIC-4 (~144 vs. ~87 ng mL^−1^) and therefore it remains plausible that in their participants, nicotine caused a depressant or relaxant effect [18, 28]. However, our results support those previously obtained when using another CNS stimulant, amphetamine, albeit by a smaller magnitude [5].

Given our results on leg extensor strength, it was surprising that NIC-2 did not affect CMJ performance. On the one hand, it might be argued that having produced more work during NIC-2 than PLA (141 ± 62 J, *p* < 0.01) could have produced greater fatigue that would mask any enhancement during the CMJ, or that the addition of velocity to force (i.e. power) attenuates any “true” effect (or single- vs. multi-joint movements). Previous studies have also found no improvement in CMJ [23] or rate of leg extensor force development [12] with smokeless tobacco; in fact, Escher et al. [12] noted an ergolytic effect when regular users consumed rather than abstained from tobacco, although this may be due to nicotine’s biphasic effect at the neuromuscular junction whereby it exerts excitatory (acutely, when naïve or after withdrawal) then inhibitory (chronically, when tolerant) effects [1, 19]. Chandler and Blair [5] also failed to demonstrate any effect of amphetamine on leg power. It might also be that a CMJ is not sufficiently sensitive a test.

### Low-Dose Nicotine Affects Pacing But Not Anaerobic Capacity During the WAnT

Previous studies have failed to detect any difference in anaerobic capacity during the WAnT when administering sublingual nicotine in naive participants [27] or smokeless tobacco in regular users [2]; therefore, in this respect, our results are in agreement. Furthermore, other CNS stimulants (caffeine, pseudoephedrine) have also failed to affect WAnT performance [7, 16]. However, that we observed a reduction in peak power yet an attenuation in fatigue when administered NIC-2 compared to PLA was surprising (Fig. 2) and difficult to explain. We observed a significant relative alkalosis immediately prior to the WAnT during NIC-2 only whereas the ~2 mmol L^−1^ higher HCO_3_^−^ was not significant (*p* = 0.2, Table 1); thus, whilst this could provide a mechanism for reduced fatigue via improved acid–base balance, it does not explain why the rate of fatigue was equally reduced during NIC-4. Indeed, as an alkaloid, nicotine is a weak base (p*K*_a_ = 8.0) that in gum form, it is buffered to alkaline pH to facilitate buccal absorption [3]. The authors feel that further explanation/discussion is not possible, especially without further mechanistic insight, as this would be too speculative.

### Potential Mechanism(s) of Action

That nicotine that had been sufficiently absorbed systemically within both NIC-2 and NIC-4 was confirmed by our measurement of cotinine concentration (Fig. 1), and that heart rate was elevated during both trials compared to PLA (Table 1). The sympatho-adrenal (excitatory) effects of nicotine are well known [14] although, given the ergogenic characteristics discussed here (muscular and anaerobic performance), it is most likely that nicotine is exerting its effects via stimulating cholinergic neurotransmission in the basal forebrain (cortical arousal) and/or enhanced mesolimbic dopaminergic activity (motivation and reward) [4]. Nevertheless, a hypoalgesic effect of nicotine should not be discounted as it has been experimentally demonstrated to increase the pain threshold [17], and might therefore prove ergogenic via antinociception as proposed by caffeine [10]. The possibility of a placebo effect cannot be discounted; indeed, it is difficult to mask the effects such as those from (m)any CNS stimulants. However, several steps were taken during the study design: (1) although only single-blind, participants were unaware of the study hypotheses and only told that they would be receiving nicotine on two of three occasions, (2) a flavour-matched placebo was used, and (3) although participants might have detected the portion of nicotine swallowed (first-pass metabolism) or the psychoactive/sympatho-adrenal effects, were a placebo effect is indeed evident in the current study then it would likely be seen for NIC-4 also and this was not the case.

### Considerations and Future Research

It is acknowledged that a sample size of *n* = 9 is relatively small; however, clear and directional statistical results were observed even with this sample. It is also worth noting that the finding of the 4 mg gum minimally affecting the performance measures was not due to side effects masking or reducing any ergogenic effect(s), as none were reported. There are many different nicotine delivery systems commonly available (over-the-counter) including nasal spray, gum, inhaler, lozenge, sublingual tablet and transdermal patch. The different delivery systems result in different nicotine bioavailability and absorption and therefore pharmacokinetics such that, for example, peak systemic concentrations are observed whilst smoking a cigarette, followed by oral snuff and chewing tobacco with gum being the lowest, possibly due to the first-pass metabolism and nicotine being retained in the gum itself [3]. The observed cotinine concentration from chewing gum was indeed lower than that observed with oral smokeless tobacco and transdermal patch [12, 24]; therefore, it would be of interest to directly compare different delivery systems on the same experimental sample and performance protocol. There is also considerable inter-individual variability in nicotine metabolism (e.g. nutrition, age, sex, medication, ethnicity, genetics; see [3]) as we observed for NIC-2 (Fig. 1) and thus, we cannot be certain that our *n* = 3 is the representative of the whole sample. Further, dosing (e.g. absolute vs. relative) needs to be more carefully considered for future studies. Finally, as the primary outcome measure of the present study was performance, future studies should include further mechanistic insights i.e. motor unit activation (twitch interpolation, electromyography).

## Conclusions

The present study has demonstrated that low-dose (2 mg) nicotine gum increases leg extensor torque, but counter-movement jump and anaerobic capacity during WAnT remained unchanged when compared to a placebo, whilst there were minimal effects of the 4-mg nicotine gum on the performance parameters measured. Together with our previous observation [24], these results indicate that nicotine per se can improve exercise endurance and muscular strength, something that WADA should continue to monitor alongside patterns of (mis)use.
